# Significant improvement in superselective intra-arterial chemotherapy for advanced paranasal sinus cancer by using indocyanine green fluorescence

**DOI:** 10.1007/s00405-013-2846-9

**Published:** 2013-12-08

**Authors:** Junkichi Yokoyama, Shinichi Ohba, Mitsuhisa Fujimaki, Masataka Kojima, Michimasa Suzuki, Katsuhisa Ikeda

**Affiliations:** 1 Department of Otorhinolaryngology-Head and Neck Surgery, Juntendo University School of Medicine, Hongo 3-1-3, Bunkyo-ku, 113-8431 Tokyo, Japan; 2 Department of Radiology, Juntendo University School of Medicine, Tokyo, Japan

**Keywords:** Paranasal sinus cancer, Indocyanine green (ICG), Superselective intra-arterial chemotherapy, CT angiography

## Abstract

Recent advances in indocyanine green (ICG) fluorescence imaging have enabled the visualization of the blood supply to tissues. For advanced head and neck cancer, intra-arterial chemotherapy has been applied for improving the prognosis and organ preservation. To identify the tumor-feeding artery, CT angiography has been shown to be useful. However, the presence of dental metals sometimes disturbs the precise evaluation of paranasal sinus cancer patients by CT angiography. The objectives of the study were to assess the feasibility of the ICG fluorescence technique during intra-arterial chemotherapy for advanced maxillary cancer. Thirty-six patients with paranasal sinus cancer who were treated by intra-arterial chemotherapy were included. Conventional CT angiography followed by 5 mg of ICG injection was performed to confirm the areas in which the drug had dispersed. Intra-arterial chemotherapy was administered at 150 mg/m^2^ of CDDP four times weekly. Additional information about the arteries feeding the tumors provided by ICG was evaluated. Out of 36 cases, in 17 (47%) the blood supply to the cancer was clearly detected by CT angiography. By adding the infrared ICG evaluation, the blood supply to the tumor was confirmed easily in all cases without radiation exposure. The information obtained from fluorescence imaging was helpful for making decisions concerning the administration of chemo-agents for paranasal sinus cancers in cases involving dental metal, or skin invasion. ICG fluorescence imaging combined with intra-arterial chemotherapy compensated for the deficiencies of CT angiography for paranasal sinus cancer. ICG fluorescence provided us clearer and more useful information about the feeders to cancers.

## Introduction

For advanced paranasal sinus cancer, which is resistant to conventional systemic chemotherapy, superselective intra-arterial chemotherapy is believed to increase the concentration of anti-cancer drugs in the tumor [1–6]. To obtain precise information about the blood supply of the tumors, we conducted CT angiography for head and neck cancer in 1998 for the first time in the world [7]. This procedure can provide accurate and detailed information about the vascular supply to head and neck cancers [1, 8–11]. However, it is difficult to confirm the drug distribution areas when the tumor is superficially invasive or the patient has undergone dental treatment with metal. When conducting intra-arterial chemotherapy for maxillary cancer, indigo carmine dye is thought to provide useful information concerning the tumor-feeding arteries [1, 2]. For deeply invasive tumors; however, conventional blue dye is not useful [1]. Furthermore, the duration for which the enhancement of the selected artery feeding can be observed is so short that it’s difficult to evaluate precisely. Recent advances in indocyanine green (ICG) fluorescence imaging have enabled visualization of the blood flow in tissues [3, 12–15]. However, the only one report using ICG technique with intra-arterial chemotherapy has been applied to oral cancer [16].We have applied this ICG fluorescence technique in combination with CT angiography for advanced paranasal sinus cancer.

The purpose of this study was to assess the feasibility of the ICG fluorescence technique during intra-arterial chemotherapy for advanced paranasal sinus cancer, especially maxillary cancer.

## Materials and methods

Thirty-six patients with paranasal sinus cancer who were treated by intra-arterial (I-A) chemotherapy concurrent to radiotherapy from April 2010 to January 2012 were included in this study. The patients’ characteristics are shown in Table 1. CT angiography was performed after the branch of a possible tumor-feeding artery was selected using conventional DSA. At the same time, 5 mg of indocyanin green (ICG) was injected and we observed whether the tumor territories were stained by using an infrared camera system (Hypereye Medical System Handy, Mizuho Ikakogyo Co. Ltd).

**Table 1:** Patients’ characteristics (TNM classification)

T/N	0	1	2b	2c	
3	3				3
4a	11	2	4	1	18
4b	12	1	1	1	16
	26	3	5	2	36

Fresh:recurrent 32:4

I-A chemotherapy was performed weekly over a 4-week period. 150 mg/m^2^ of CDDP was administered superselectively through feeding arteries at 5 mg/min. Sodium thiosulfate at a dose of 200-fold that of the CDDP was injected concurrently intravenously to neutralize the adverse effects of CDDP.

We used both a retrograde approach via a temporal artery and a femoral artery. I-A chemotherapy was performed via the femoral artery when the tumor invaded the contralateral side or when contralateral side lymph node metastasis occurred. When the targeted artery had many branches that were not blood supplies to the cancer, we used a microcatheter through a 5 Fr guide catheter positioned inside the targeted artery.

We evaluated the diagnostic sensitivity of CT angiography and ICG fluorescence technique. Furthermore, when the tumor extended beyond the midline, the drug distribution could be made to extend to the contralateral tumor by manual compression of the contra-carotid artery, confirmed using the ICG fluorescence technique. Informed consent was obtained from each patient before treatment and the study was approved by the Human Ethics Review Committee of Juntendo University.

## Results

Thirty-six patients with advanced paranasal sinus cancer received definitive chemoradiation with I-A chemotherapy. These advanced cases were not suitable for surgical treatment for organ preservation. Superselective I-A chemotherapy via a superficial temporal artery was performed in 18 patients. We initially carried out superselective I-A chemotherapy via the femoral artery twice in 3 cases of N2c and 15 cases of T4, in which the contralateral nasal or paranasal sinus was invaded by cancer. After this procedure, the patients were treated by superselective I-A chemotherapy via a superficial temporal artery carried out twice. There were no significant complications. Table 2 shows a list of the infused arteries. The total number of I-A chemotherapies was 164. The mean I-A chemotherapy was 4.56 (range 3–6). The total number of superselectively infused arteries was 413. Of the 413 infused arteries, the numbers of infused maxillary arteries, facial arteries, transverse facial arteries, and internal carotid arteries were 164, 79, 64, and 72, respectively.

**Table 2:** The summary of infused arteries

Infused artery	Maxillary A	Facial A	Transverse facial A	Superficial temporary A	Occipital A
Total	164	79	64	12	10
Average	4.56	2.19	1.8	0.33	0.28
Range	2–6	0–5	0–5	0–5	0–2

Infused artery	Internal carotid A	Posterior auricular A	Contralateral maxillary A	Contralateral internal carotid A	Ascending pharyngeal A	Total
Total	72	2	6	2	2	413
Average	2	0.06	0.17	0.06	0.06	
Range	0–6	0–2	0–4	0–2	0–2	

**Fig. 1:**
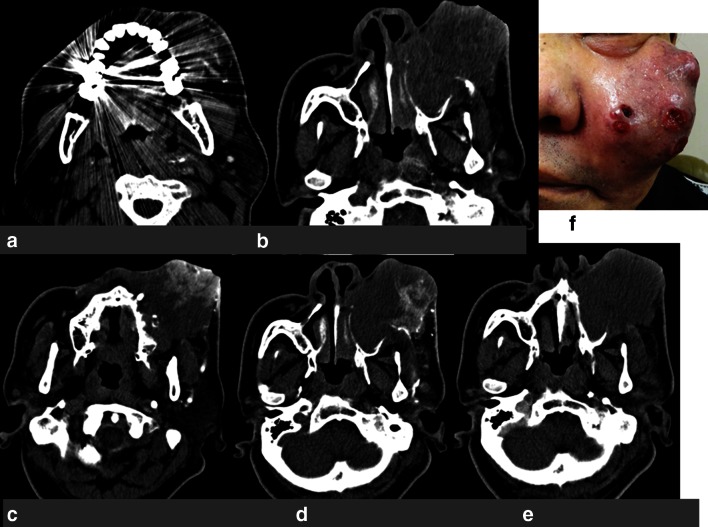
Case 1: a 66-year-old man with maxillary cancer (T4AN2bM0). **a** CT angiography obtained in the selected left side maxillary artery. It was difficult to confirm the vascular territory due to dental metals. **b** CT angiography obtained in the selected left side maxillary artery. It was difficult to confirm the vascular territory due to obstacle enhancement. **c** CT angiography obtained in the selected left side facial artery. It was sufficiently clear to confirm the vascular territory. **d** CT angiography obtained in the selected left side transverse facial artery. It was sufficiently clear to confirm the vascular territory. **e** CT angiography obtained in the selected left side internal carotid artery. It was difficult to confirm the vascular territory due to obstacle enhancement. **f** Maxillary cancer invading the face before treatment

**Fig. 2:**
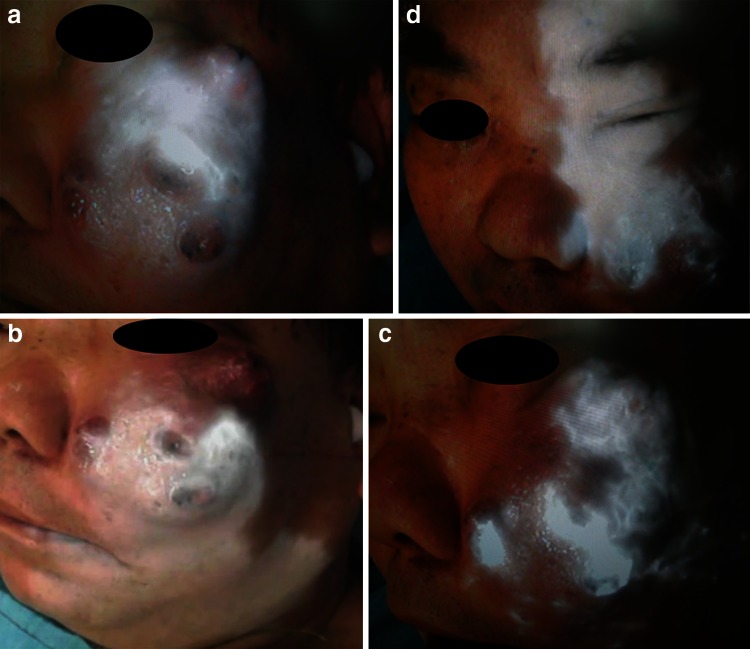
ICG fluorescence imaging. **a** ICG fluorescence imaging of the left maxillary artery. **b** ICG fluorescence imaging of the left facial artery. **c** ICG fluorescence imaging of the left transverse facial artery. **d** ICG fluorescence imaging of the left internal carotid artery. The cancer involving the facial skin was clearly visualized under fluorescent imaging of each vascular area

**Fig. 3:**
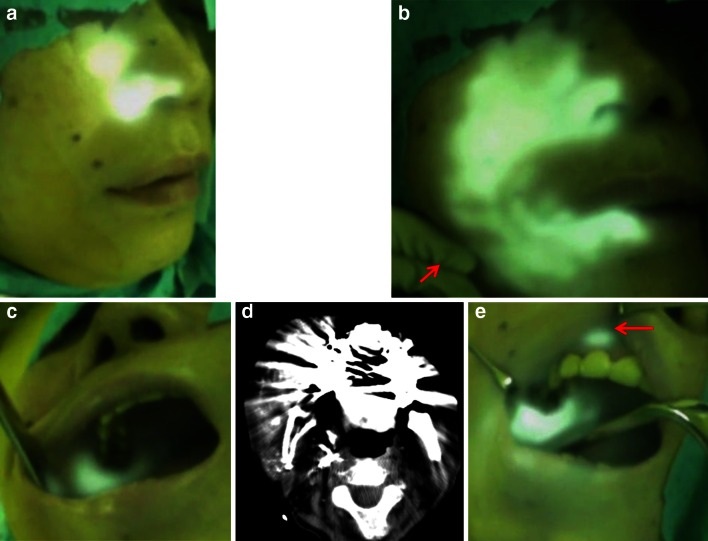
Case 2: a 60-year-old woman with maxillary cancer (T4aN0M0), which extended to the oral cavity and cheek with communicating branches between the maxillary artery and facial artery. **a** The right cheek by ICG fluorescence imaging at the right maxillary artery. **b** The right cheek by ICG fluorescence imaging with right manual facial artery compression. The ICG fluoresced areas extended throughout the maxillary artery and the facial artery was infused at the right maxillary artery with right manual facial artery compression (*arrow*). **c** The oral cavity by ICG fluorescence imaging at the right maxillary artery. **d** CT angiography obtained in the right maxillary artery. It was difficult to confirm the vascular territory due to dental metal. **e** The oral cavity by ICG fluorescence imaging with right manual facial artery compression. The ICG fluoresced oral cavity extended throughout the maxillary artery and the facial artery with right manual facial artery compression

CT angiography revealed the vascular territories of selected arteries in only 17 cases (47 %). The reasons for the failure in detecting the tumor-feeding arteries included dental metals (10 cases) and mucosal or skin invasions (superficially invasive tumor) (8 cases). Additionally, CT angiography could not detect communicating branches between the feeders (7 cases).With the infrared ICG evaluation, the arteries supplying the tumors were confirmed accurately in all cases (Figs. 1, 2). In the cases with a tumor invading the cheek or facial skin, the use of communicating branches of the maxillary artery for drug delivery to the whole tumor was confirmed by manual compression of the ipsilateral facial artery (Fig. 3). The alteration of blood flow by manual facial artery compression could be directly observed using the ICG fluorescence technique (Fig. 3). In the case of a tumor crossing the midline, drug delivery to the whole tumor was confirmed by manual compression of the contralateral carotid artery, or maxillary artery. The alteration of blood flow by manual contralateral artery compression was directly viewed using the ICG fluorescence technique (Fig. 3).

**Fig. 4:**
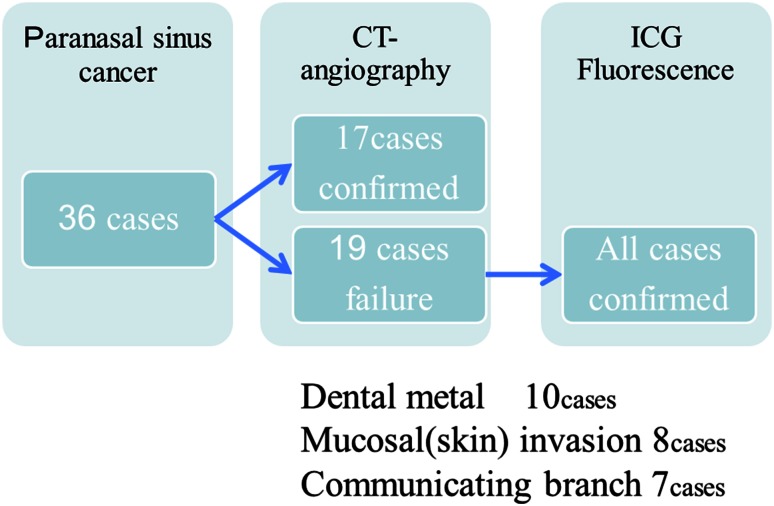
The confirmation rate of the tumor-feeding arteries with CT angiography and ICG fluorescence imaging

Confirmation of the tumor-feeding arteries was established using CT angiography and ICG fluorescence imaging, as shown in Fig. 4.

The effect of I-A chemotherapy was that CR and PR were 86 and 14 %, respectively.

**Fig. 5:**
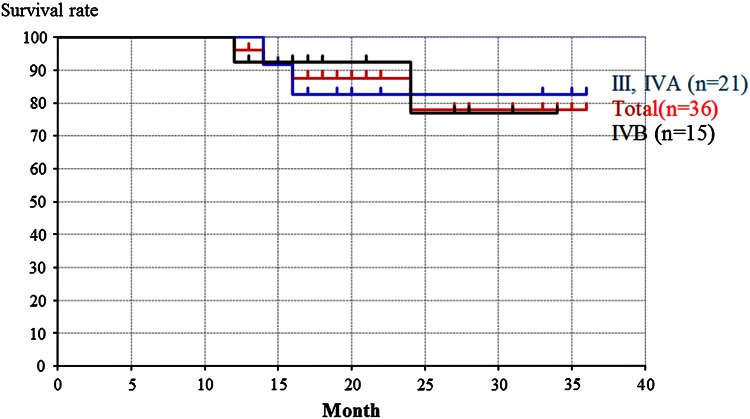
The overall survival rates

Of the definitive chemoradiation group, the overall survival rates of the cases, stage III and IVA group, and stage IVB group were 78, 82, and 77 %, respectively (Fig. 5). The difference between the overall survival rate of the stage III and IVA group and the overall survival rate of the stage IVB group was not significant (Fig. 5).

## Discussion

Chemoradiotherapy for head and neck cancer plays an important role in organ preservation, but there are many cases such as paranasal sinus cancer for which conventional systemic chemotherapies do not work at all. CDDP is the most promising drug for head and neck cancer. When high doses of CDDP are used, various adverse effects can be observed, such as gastrointestinal toxicity, renal toxicity, and hematotoxicity. For such chemo-resistant cancers including paranasal sinus cancer, superselective intra-arterial chemotherapy is considered to increase the concentration of the anti-cancer drug in the cancer tissue, exerting powerful effects on the primary cancer [1–6]. This procedure is reportedly capable of achieving a positive prognosis as well as good organ preservation.

To achieve an effective therapeutic result for paranasal sinus cancer with intra-arterial chemotherapy, precise evaluation of the tumor-feeding artery and drug distribution territories is required. Digital subtraction angiography (DSA) is applied for all cases of I-A chemotherapy; however, DSA cannot clearly detect the border between the oral mucosa and surface invasion tumor. CT angiography clearly detects the border between the normal paranasal sinus and deeply invasive cancer by using three-dimensional sections. Therefore, CT angiography in addition to DSA has provided more precise identification of the blood supply to the tumor [1, 7, 8]. However, we are sometimes not able to confirm the tumor-feeding artery in paranasal sinus cancer patients with dental metal fillings or when the tumor has spread to oral cavities or superficially to the facial skin. Furthermore, repeated CT angiography increases the X-ray exposure, which is a significant problem not only for patients but also for the medical staff, especially when a manual carotid compression technique is applied. Previously, indigo carmine dye was often used to confirm the blood supply to the tumor. However, the stain disappears soon after the injection and cannot be observed in cases of deeply invasive tumors [1, 2].

Recently, the ICG fluorescence technique was developed and has been used in various fields [3, 12–15]. The excitation and emission profiles for ICG lie in the near-infrared wavelengths, which allow penetration and imaging of vessels below a few millimeters of tissue [12]. It provides visualization of the blood supply to reconstructed organs, and sentinel lymph nodes in cancer surgery including head and neck cancer surgery [3, 13, 15, 17]. ICG fluorescence has been used for navigation surgery and intraoperative detection of cancers [12, 18, 19].

We have reported that the ICG fluorescence technique can be a very useful method for treating oral cancers with I-A chemotherapy in patients with dental metal [16].

In this study, we also report our success in identifying the tumor-feeding arteries in paranasal sinus cancer by ICG fluorescence imaging. We found that the ICG fluorescence technique was a very useful method even in patients with dental metal. For tumors with multiple feeding arteries, ICG fluorescence in selectively infused arteries could be evaluated clearly and lucidly. Accordingly, we were able to confirm that the whole tumor was covered and infused with the anti-cancer drug.

We sometimes performed the I-A infusion chemotherapy with manual compression of the contralateral facial artery or lingual artery in cases of oral cancer with tumors spreading to the contralateral oral cavity. This enabled alteration of the blood flow and the area supplied by the contralateral facial, or lingual artery was sufficiently confirmed to be covered by using the ICG fluorescence technique. In the present study, we sometimes perform the I-A infusion chemotherapy with manual compression of the ipsilateral facial artery in cases of advanced paranasal sinus cancers with tumors spreading to the oral cavity or facial skin. This enables alteration of the blood flow and the area supplied by the ipsilateral facial artery is sufficiently identified to be covered by using the ICG fluorescence technique. The changes of the blood flow were directly observed using the ICG fluorescence technique and effective I-A chemotherapy for the affected side alone could be conducted safely. No complication resulted from compression of the carotid artery, or facial artery. However, the same manual compression procedures during CT angiography would involve too high a radiation exposure if conducted each time. In this paper, we emphasize the following points concerning oral cancers: the drug delivery to the whole tumor crossing the midline from the contralateral side was confirmed directly by using the ICG fluorescence technique (horizontal communication). This is because there are communicating branches to the lateral sides in oral cancer. However, in the present advanced paranasal sinus cancers, communicating branches develop vertically, such as the maxillary artery communicating to branches of the facial artery (vertical communication). As a result, the significance of this method differs significantly between advanced oral cancer and advanced paranasal sinus cancer. Because one of most problematic issues of using I-A chemotherapy for advanced paranasal sinus cancers is that the tumors are often supplied by the internal carotid artery and consequently, to prevent brain complications, I-A chemotherapies were previously not indicated for such cases.

Recently, the use of ICG fluorescent image has enabled us to clearly detect tumor staining and reveal communicating branches between the internal carotid artery and maxillary artery, facial artery, and transverse facial artery (vertical communication). As a result, this ICG fluorescence imaging procedure, when combined with the technique for altering the blood flow, has enabled us to safely treat advanced paranasal sinus cancer, while improving the prognosis and preserving organ and function.

Additionally, ICG fluorescence is a useful method for confirming the arterial blood supply to paranasal sinus cancers because of the high excitation of ICG and deep penetration of the tissue to approximately 10 mm. As a result, we can use superselective I-A chemotherapy effectively for most paranasal sinus cancers that have invaded less than 10 mm. However, tumors invading more deeply than 10 mm require other imaging methods, such as simultaneous MRI angiography. Once the stained field of each feeding artery has been detected in cases of tumors invading the base of the skull or face by CT angiography, the ICG fluorescence technique can be used to compensate for defects in the CT angiography by identifying the feeding artery precisely and safely. It should be noted that a fine and sensitive endoscope for ICG fluorescence imaging is required for the detection of nasal cavity cancer. This technique is feasible and gives new promising options for advanced paranasal sinus cancers. Further investigations can lead to the development of a new minimally invasive multimodal therapy targeting advanced paranasal sinus cancers in the near future.

## Conclusion

ICG fluorescence imaging for intra-arterial chemotherapy revealed the blood supplies to paranasal sinus cancers more accurately than CT angiography, especially in cases of superficial spread or those with dental metal. The application of ICG fluorescence together with CT angiography provides more accurate information about the feeding arteries to tumors and enables effective intra-arterial chemotherapy, while avoiding complications.

## Abbreviations

ICG: Indocyanine green
NIR: Near-infrared
I-A: Intra-arterial
CT: Computed tomography
DSA: Digital subtraction angiography
